# Spatial Distribution and Determinants of Early Marriage among Married Women in Ethiopia: A spatial and Multilevel Analysis

**DOI:** 10.1186/s12905-020-01070-x

**Published:** 2020-09-15

**Authors:** Adugnaw Zeleke Alem, Yigizie Yeshaw, Sewnet Adem Kebede, Alemneh Mekuriaw Liyew, Getayeneh Antehunegn Tesema, Chilot Desta Agegnehu, Achamyeleh Birhanu Teshale

**Affiliations:** 1 Department of Epidemiology and Biostatistics, Institute of Public Health, College of Medicine and Health Sciences, University of Gondar, Gondar, Ethiopia; 2 Department of Physiology, School of Medicine, College of Medicine and Health Sciences, University of Gondar, Gondar, Ethiopia; 3 School of Nursing, College of Medicine and Health Sciences and Comprehensive Specialized Hospital, University of Gondar, Gondar, Ethiopia

**Keywords:** Spatial distribution, Multi-level analysis, Early marriage, Ethiopia

## Abstract

**Background:**

Early marriage is a global public health problem that is mainly practiced in South Asia, Latin America, and sub-Saharan Africa including Ethiopia. It raises the risk of early childbearing of women, higher rates of divorce, and an increased risk of maternal and child death. However, little is known about the spatial distribution and determinants of early marriage in Ethiopia. Therefore, this study aimed to assess the spatial distribution and determinants of early marriage among ever-married women in Ethiopia.

**Methods:**

A detailed analysis of the 2016 Ethiopian Demographic and Health Survey data was conducted. A total weighted sample of 11,646 reproductive-age married women were included in the analysis. To identify significant hotspot areas of early marriage the Bernoulli model was fitted using SaTScan version 9.6 software. Additionally, to explore the spatial distributions of early marriage across the country ArcGIS version 10.1 statistical software was used. For the determinant factors, the multilevel logistic regression model was fitted. Deviance was used for model comparison and checking of model fitness. In the multivariable multilevel analysis, Adjusted Odds Ratio (AOR) with 95% CI was used to declare significant determinants of early marriage.

**Results:**

The finding of this study revealed that the spatial distribution of early marriage was significantly varied across the country with Global Moran’s I = 0.719 and p value < 0.001. The primary clusters were detected in Tigray, Amhara, and Afar regions. Both individual and community-level factors were associated with early marriage. Having no formal education (AOR = 4.25, 95% CI 3.13–5.66), primary education (AOR = 3.37, 95% CI 2.80–4.92), secondary education (AOR = 1.75, 95% CI 1.32–2.33), and a decision made by parents (AOR = 1.88, 95% CI 1.68–2.09) were individual-level factors associated with higher odds of early marriage. Among the community-level factors, the region was significantly associated with early marriage. Thus, living in Afar (AOR = 1.82, 95%CI 1.37–2.42), Amhara (AOR = 1.77, 95% CI 1.38–2.77), and Gambela (AOR = 1.44, 95% CI 1.09–190) was associated with higher odds of early marriage. Whereas, living in Addis Ababa (AOR = 0.50, 95% CI 0.36–0.68) was associated with a lower chance of early marriage.

**Conclusion:**

The spatial distribution of early marriage was significantly varied in Ethiopia. Women’s education, women’s autonomy, and region were found to be the significant determinants of early marriage. Therefore, public health interventions targeting those identified significant hotspot areas of early marriage are crucial to reduce the incidence of early marriage and its consequence. In addition, enhancing women's education and empowering them to make their own choices are vital for changing the customs of the community and eliminating early marriage in Ethiopia.

## Background

According to the United Nations Children's Fund (UNICEF) definition, early marriage is defined as marriage occurred while younger than 18 years of age [1]. Age at marriage is a period of transition to adulthood, the point at which certain options in education, employment, and participation in society are foreclosed; and the beginning of regular socially acceptable time for sexual activity and childbearing [2]. Globally, more than 700 million women are married before their 18^th^ birthday [3]. Early marriage is a significant health and children’s rights concern in many low- and middle- income countries [4]. Of the global prevalence of early marriage, more than half is practiced in South Asia, Latin America, and Sub-Saharan Africa [5, 6]. Despite programmatic and legislative efforts to stop child marriage, still it is a common problem in Sub-Saharan Africa [7], which affects 54% of women aged 20–24 years with large disparities among countries [6, 8, 9].

The practice of early marriage is also common among Ethiopian women. The prevalence of early marriage among reproductive-age group women ranges from 26% in Addis Ababa to 87% in the eastern region of Amhara [3, 10, 11]. In addition, according to the reports from Ethiopian Demographic and Health Surveys (EDHS) 2005, 2011, and 2016, the prevalence of early marriage among women aged 20–24 years is 66%, 63%, and 58% respectively [12–14].

Early marriage is associated with an increased risk of early childbearing of a mother, low economic status of women, termination of education, risk of sexually transmitted infection, higher rates of divorce, a number of poor social and physical outcomes for young women, and their offspring, complications of pregnancy and an increased risk of death for the mother and their child [15–17].

Different factors have been associated with early marriage among women. These include: education status of women and parents, number of family members, residence, economic status of households [18–22], decision on first marriage [23], religion [2], knowledge about the best marital age, region [2], knowledge about accusing of early marriage [18], and media exposure [4].

Though ending an early marriage is one of the prioritized agenda of the Sustainable Development Goals, investments to end the practice remain limited across the globe [24], indicating that more should be done to alleviate this problem. Additionally, there is a scarcity of information on the effect of community-level determinants on early marriage, Therefore, this study aimed to explore the spatial distribution and determinants of early marriage among women in Ethiopia. Assessing geographic variations and determinants of early marriage is essential to understand where the practice is common, the factors that drive it, and evaluating the effectiveness of efforts made to eliminate the problem.

### Conceptual framework

**Fig. 1:**
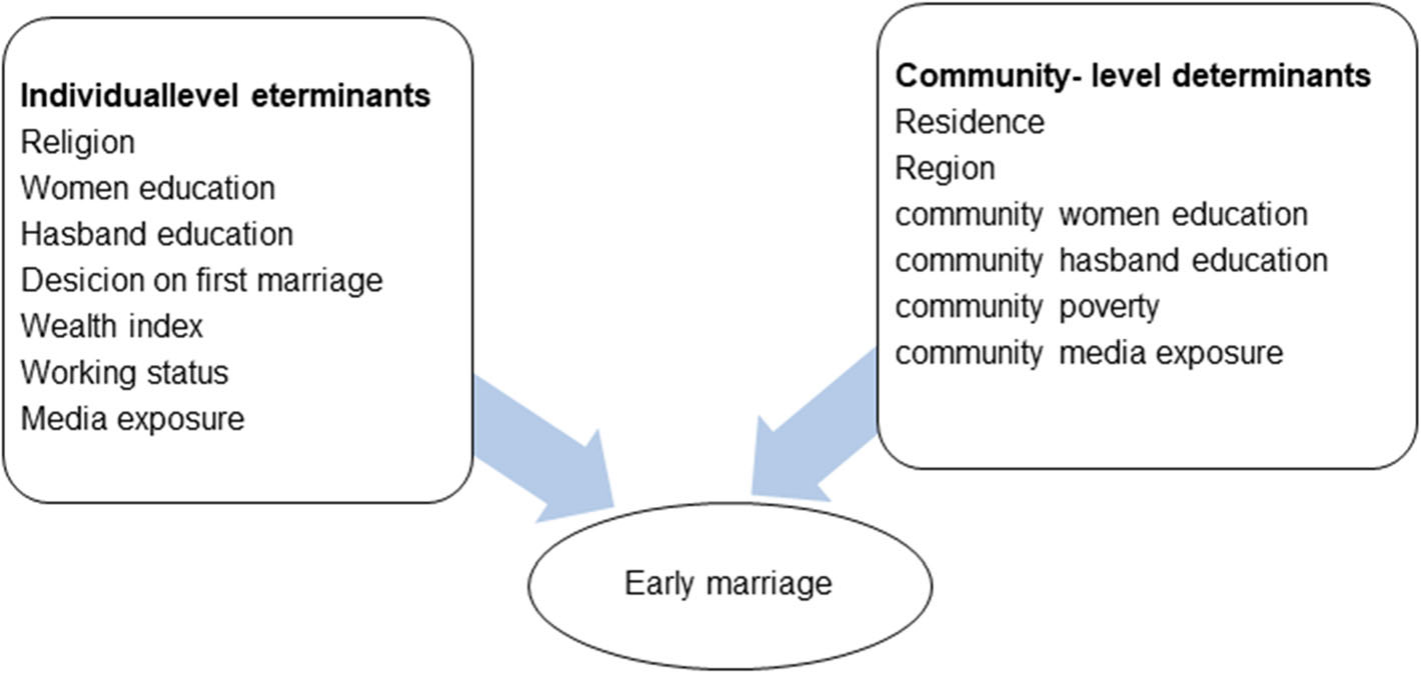
Conceptual framework on determinants of early marriage among married Ethiopian women

The conceptual framework presented in Fig. 1, indicates the relationship between early marriage and independent variables. The independent variables adapted from different pieces of literature include both individual-level factors (women’s level of education, religion, education level of husband, working status, type of media exposure, wealth index and decision-making power on first marriage) and community-level factors (community women education, community husband education, community poverty level, community level of media exposure, residence, and region) [2, 4, 18–23].

## Methods

### Study design and area

We used the EDHS 2016 data to identify factors and the extent of spatial patterns of early marriage in Ethiopia. Ethiopian Demographic and Health Survey 2016 was a recent population-based cross-sectional survey conducted across the country. Ethiopia found at the Horn of Africa (3°-14^o^ N and 33°-48°E). The country covers 1.1 million Square km and it has a high central plateau that varies from 4550 m above sea level down to the Afar depression to 110 m below sea level. Administratively, Ethiopia is federally decentralized into nine regions and two city administrations and regions are divided into 68 zones, and zones, into administrative units called districts (817). Each district is further subdivided into the 16,253 lowest administrative unit, called kebele.

### Data source and population

The EDHS is a survey designed to provide population and health indicators at the national and regional levels. It is collected every five years. For this study, we used the fourth EDHS 2016, the recent EDHS. A stratified two-stage cluster sampling procedure was employed for the survey. In the first stage, a total of 645 enumeration areas (202 in urban areas) were selected using systematic sampling with probability proportional to size. In the second stage, a fixed number of 28 households per cluster were selected randomly from the household listing. Data were collected using a structured, interviewer-administered questionnaire. To ensure data quality, questionnaires were pretested, training was given for both data collectors and supervisors [25]. The study population for this study was all women aged from 15–49 years across all regions of Ethiopia. A total weighted sample of 11,646 ever-married reproductive-age women were included for the final analysis.

### Variables of study

#### Outcome variable

The outcome variable in this study was early marriage which refers to marriage before 18 years of age. It was a binary outcome variable coded 0 as “No” and 1 as “Yes”.

#### Independent variables

For this study, we included both individual and community-level factors that are associated with early marriage.

#### Individual-level variables

Individual-level variables were: women’s level of education (categorized as no education, primary, secondary, and higher), religion (recoded as Muslim, orthodox, Protestant, and Catholic), education level of husband (categorized as no education, primary, secondary, and higher), working status (recoded as not working and working), type of media exposure (labeled as 0 if there is no media exposure at all, 1 if there is media exposure to either of radio, newspaper, television or internet; 2 if there is an exposure to two of media types; 3 if there is an exposure to three of media types and 4 if there is a exposure to all of media types), wealth index (categorized as poorest, poorer, middle, richer, and richest), and decision-making power on first marriage (categorized as myself, parents, and relatives/others).

#### Community-level variables

The community-level variables included in our study were region, place of residence, and other variables obtained by aggregating individual-level variables which include: community women’s education is defined as the proportion of women with a minimum of primary level of education, community husband education is defined as the proportion of husband with a minimum of primary level of education, community poverty level is measured as the proportion of women in the poorest and poorer quintile derived from data on wealth index, and community media exposure is defined as the proportion of women exposed to at least type of media; radio, newspaper television, and internet. Each aggregated community variables were categorized into low and high based on their national median value, because they were not normally distributed.

### Statistical analysis

#### Spatial analysis

ArcGIS version 10.1 and SaTScan version 9.6 software were used for the spatial analysis. The spatial autocorrelation (Global Moran’s I) statistic measure was used to evaluate whether the spatial distribution of early marriage was dispersed, clustered, or randomly distributed in Ethiopia. Moran's I is a spatial statistics used to measure spatial autocorrelation by taking the entire data set and produce a single value which ranges from -1 to + 1. A positive value for Moran’s Index indicates a clustered pattern of early marriage, while a negative value indicates a dispersed pattern and Moran’s I value close to zero indicates random distribution of early marriage [26, 27].

Hot spot analysis (Getis-OrdGi* statistic) was computed by calculating GI* statistic for each area and statistical outputs with high GI* indicates, “hotspot” areas and low GI* indicates “cold spot” areas.

Using the Kuldorff’s SaTScan version 9.6 program, spatial scan statistical analysis was used to classify statistically important hotspots areas. To fit the Bernoulli model, women who got married before the age of 18 years (those who have early marriage) were taken as cases and women who got married at the18 years and above were taken as controls. The numbers of cases in each location have Bernoulli distribution and a maximum spatial cluster size of < 50% of the population was used as an upper limit. Z-score is computed to determine the statistical significance of clustering, and the p-value was used to determine if the number of observed early marriage within the potential cluster was significant or not. The null hypothesis of no clusters was rejected when the p-value ≤ 0.05. Based on 999 Monte Carlo replications the significant clusters were identified and ranked based on their likelihood ratio test.

In addition, the spatial interpolation technique was applied to predict the magnitude of early marriage on the unsampled areas based on the values observed in the sampled clusters. In this study, the ordinary Kriging interpolation technique was used to predict early marriage in unobserved areas of Ethiopia (it had the lowest residual and root mean square error as compared to others).

#### Multilevel analysis

The data were analyzed using STATA version 14 software. Sampling weight was done before doing any statistical analysis, to adjust for the non-proportional allocation of the sample to different regions and their urban and rural areas as well as to adjust for the non-response rates. Due to the hierarchical nature of the DHS data, we used a multilevel logistic regression model, in which we fitted four models. We first fit an empty model (a model with no independent variables) to check the variability of early marriage in the community and provide evidence to assess random effect using the Intra-class Correlation Coefficient (ICC). Then model 2 (a model with only the individual-level variables) and model 3 (a model with only community-level variables) were performed separately. Finally, model 4 (a model with both individual and community-level variables were simultaneously included.

To observe random-effects Median Odds Ratio (MOR), ICC, and Proportional Change in Variance (PCV) were calculated. The ICC was calculated by dividing cluster level variance (VA) by the individual-level variance (VB) plus 3.29 (π^2^/3), i.e.[VA/(VB + 3.29)] [28]. Median Odds Ratio was calculated as follows; exp.[√(2 × VA) × 0.6745]. During this formula, VA is the cluster level variance and 0.6745 is the value from the 75^th^ percentile of the cumulative distribution function of the normal distribution with mean = 0 and variance = 1 [28, 29]. The following equation was used to estimate the PCV; [(VA-VB)/VA]*100, where; VA is community variance of model without covariates (model 1) and VB is community variance in the models including individual (model 2), community (model 3) or both individual and community-level covariates (model 4) [28]. The appropriate model was selected using Deviance. Based on this selection criteria, the model with the lowest deviance (model 4) was considered to be a better model to fit the data.

Both bi and multivariable multilevel logistic regression models were fitted to identify factors that affect early marriage. All variables with a *p*-value < 0.20 at bi-variable multilevel analysis were entered into the multivariable multilevel analysis. Finally, adjusted odds ratio (AOR) with their corresponding a 95% confidence interval was determined and those variables with *p*-value < 0.05 in the multivariable analysis were considered as significant factors that were associated with early marriage.

## Results

### Background characteristics of respondents

A total of 11,646 women were included in this study. Of these, 6267 (66.0%) of women’s living in a rural area were married before 18 years old. Three-fourth (75.0%) of the participants from Afar and 73.0% of participants from Amhara regions were married before the age of 18 years. More than two-thirds (69.6%) of women with no formal education was married before their 18^th^ birthday **(Table ****1****).**

**Table 1:** Background characteristics of Respondents in Ethiopia, EDHS 2016

Variables	Early marriage (*N* = 11,646)	
	**Yes (N, %)**	**No (N, %)**
**Residence**		
Rural	6297 (66.0)	3247 (34.0)
Urban	1025 (47.8)	1077 (52.2)
**Religion**		
Muslim	2532 (64.8)	1374 (35.2)
Orthodox	3158 (63.5)	1812 (36.5)
Protestant	1471 (58.9)	1027 (41.1)
Catholic	37 (44.6)	48 (55.4)
Others	122 (65.2)	65 (34.8)
**Working status of respondents**		
Not working	4959 (64.2)	2764 (35.8)
working	2363 (60.2)	1559 (39.8)
**Level of women education**		
No education	4910 (69.6)	2148 (30.4)
Primary	2039 (60.9)	1311 (39.1)
Secondary	267 (34.9)	497 (65.1)
Higher	106 (22.5)	366 (77.5)
**Husband education**		
No education	3188 (68.1)	1496 (31.9)
Primary	2449 (64.9)	1323 (33.1)
Secondary	467 (47.9)	508 (52.1)
Higher	241 (33.8)	471 (66.2)
**Wealth Index**		
Poorest	1456 (65.6)	763 (34.4)
Poorer	1587 (69.5)	697 (30.5)
Middle	1530 (65.9)	793 (34.1)
Richer	1405 (62.7)	837 (37.3)
Richest	1343 (52.1)	1233 (47.9)
**Decision on 1**^**st**^** marriage**		
Myself	1985 (48.5)	2110 (51.5)
Parents	5016 (71.0)	2053 (29.0)
Relatives/Others	108 (63.1)	63 (36.9)
Region		
Tigray	543 (64.1)	304 (35.9)
Afar	81 (75.0)	27 (25.0)
Amhara	2107 (73.0)	778 (27.0)
Oromia	2751 (62.1)	1682 (37.9)
Somali	200 (55.9)	158 (44.1)
Benishang	81 (64.8)	44 (35.2)
SNNPR	1343 (58.1)	967 (41.9)
Gambela	22 (64.7)	12 (35.3)
Harari	16 (55.2)	13 (44.8)
Addis Ababa	141 (31.3)	310 (68.7)
Dire Dawa	34 (55.7)	27 (44.3)
Type of media exposure		
0	4723 (65.9)	2448 (34.1)
1	1460 (64.2)	815 (35.8)
2	887 (58.9)	620 (41.1)
3	231 (40.7)	336 (59.3)
4	22 (17.5)	104 (82.5)
Community women education		
Low	2892 (56.5)	2228 (43.5)
High	4430 (67.9)	2096 (32.1)
Community husband education		
Low	3226 (58.0)	2333 (42.0)
High	4096 (67.3)	1990 (32.7)
Community poverty		
Low	3763 (59.3)	2586 (40.7)
High	3559 (67.2)	1738 (32.8)
Community media exposure		
Low	448 (16.1)	2329 (83.9)
High	3874 (66.0)	1994 (34.0)

### Spatial distribution of early marriage

**Fig. 2:**
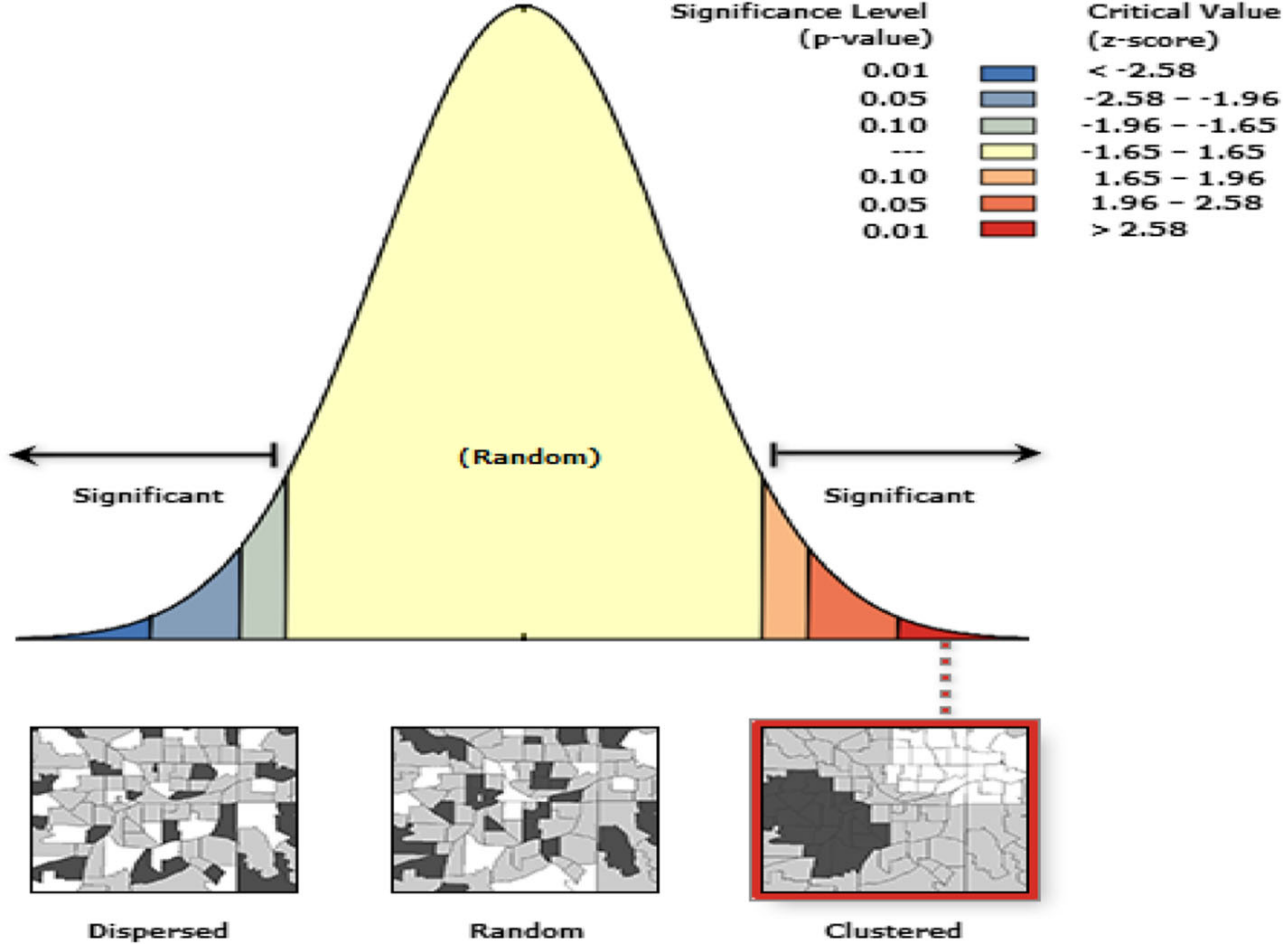
Spatial autocorrelation based on feature locations and attribute values using the Global Moran’s I statistic

**Fig. 3:**
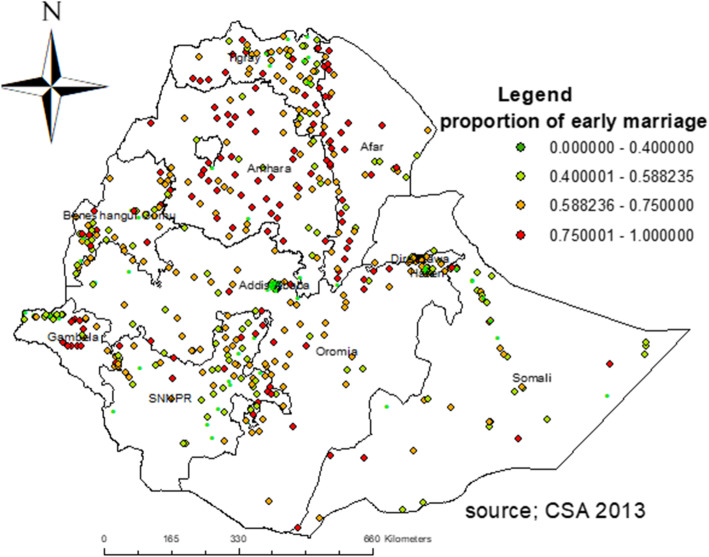
Spatial distribution of early marriage among women in Ethiopia using 2016 EDHS data

The early marriage was significantly varied across the country (Global Moran’s I = 0.719, p- value < 0.001) (**Fig. ****2**). The highest prevalence of early marriage was observed in the Amhara, Afar, and Central parts of the Gambela regions. On the other hand, Addis Ababa, eastern SNNPR, and northern Gambela had the lowest prevalence of early marriage (Fig. 3).

#### Hot spot and cold spot analysis

**Fig. 4:**
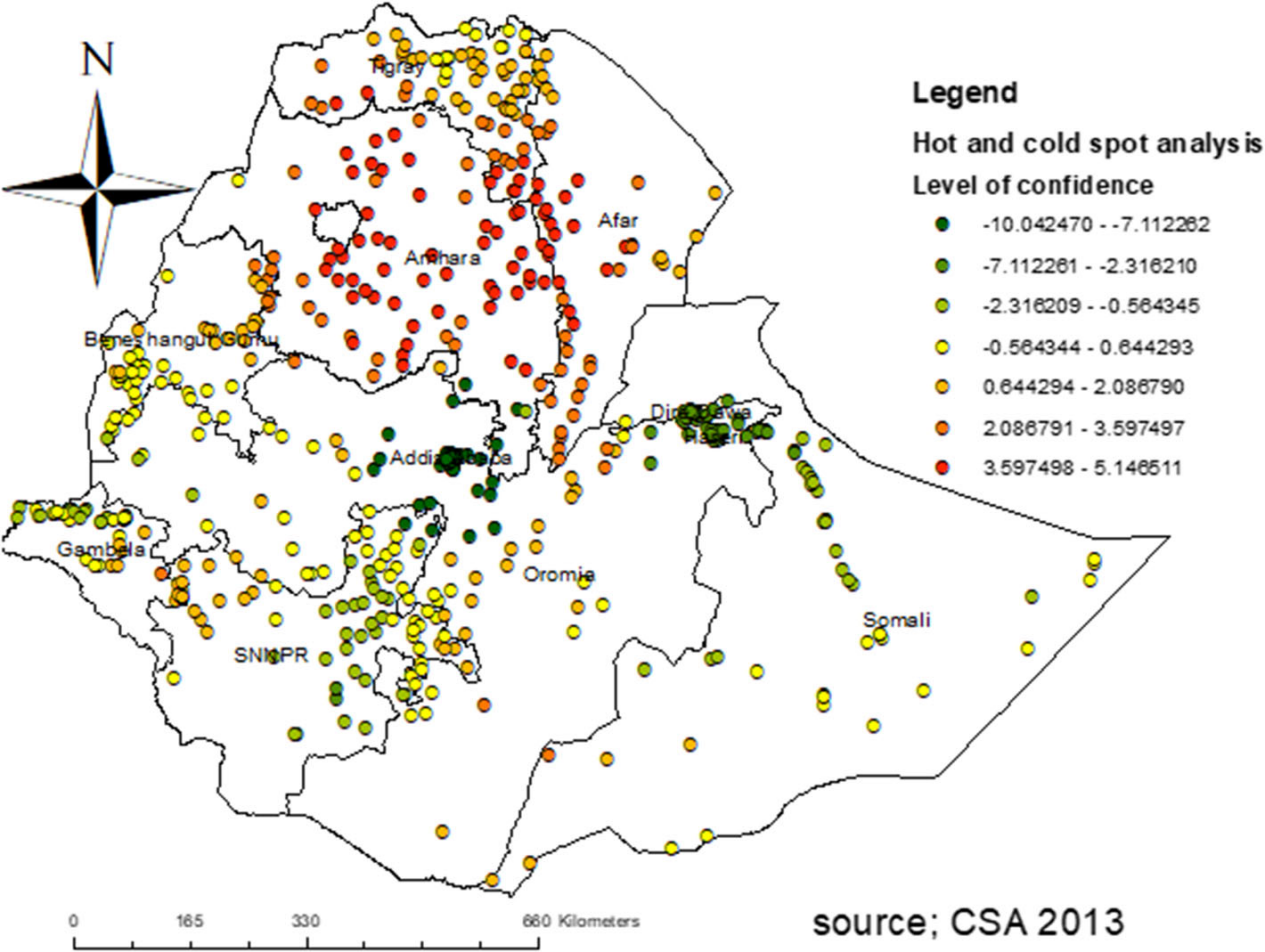
Hotspot analysis of early marriage among women in Ethiopia, EDHS 2016

In the hotspot analysis, the significant hotspot areas of early marriage were identified in the Amhara, Afar, southwestern Gambela, and southern Tigray regions whereas the significant cold spot areas were located in Addis Ababa, Dire Dawa, eastern SNNPR, and northwestern Gambela regions (**Fig. ****4**).

#### Spatial scan statistical analysis

**Fig. 5:**
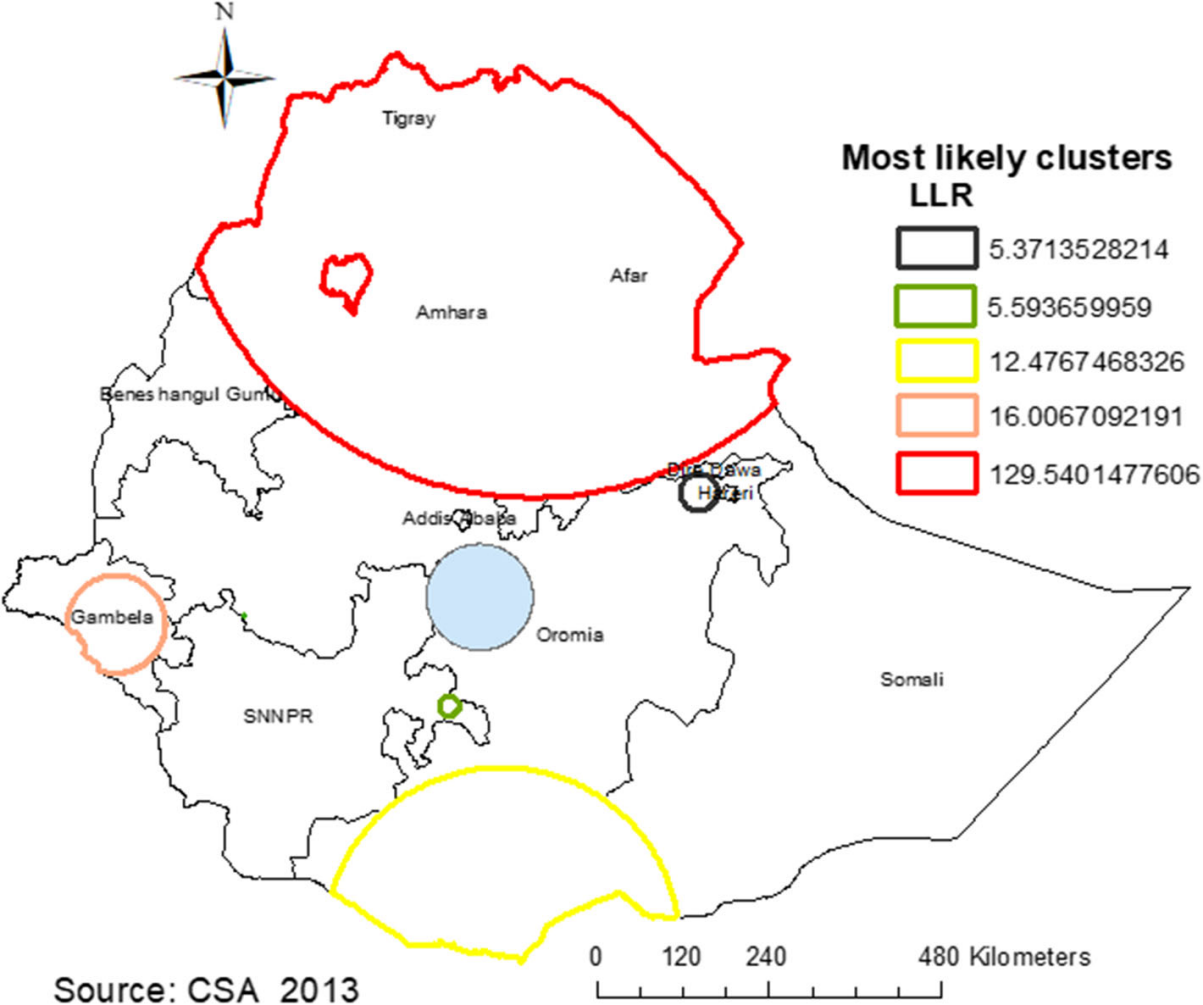
Sat Scan analysis of early marriage among women in Ethiopia, EDHS 2016

The spatial scan statistical analysis identified a total of 174 significant clusters of early marriage. Of these, 155 clusters were primary clusters (LLR = 129.54, RR = 1.28, P < 0.001) which were located in Amhara, Tigray, and Afar regions. The scanning window for these most likely clusters was centered at 13.720700 N, 39.700094 E with 496.67 km radius. This finding indicates that women within the spatial window were 1.28 times more likely to get married before 18 years as compared to women outside the spatial window. Whereas, the secondary significant clusters of early marriage were identified in Gambela and the southern part of Oromia regions (**Fig. ****5**).

### Kriging interpolation of early marriage

**Fig. 6:**
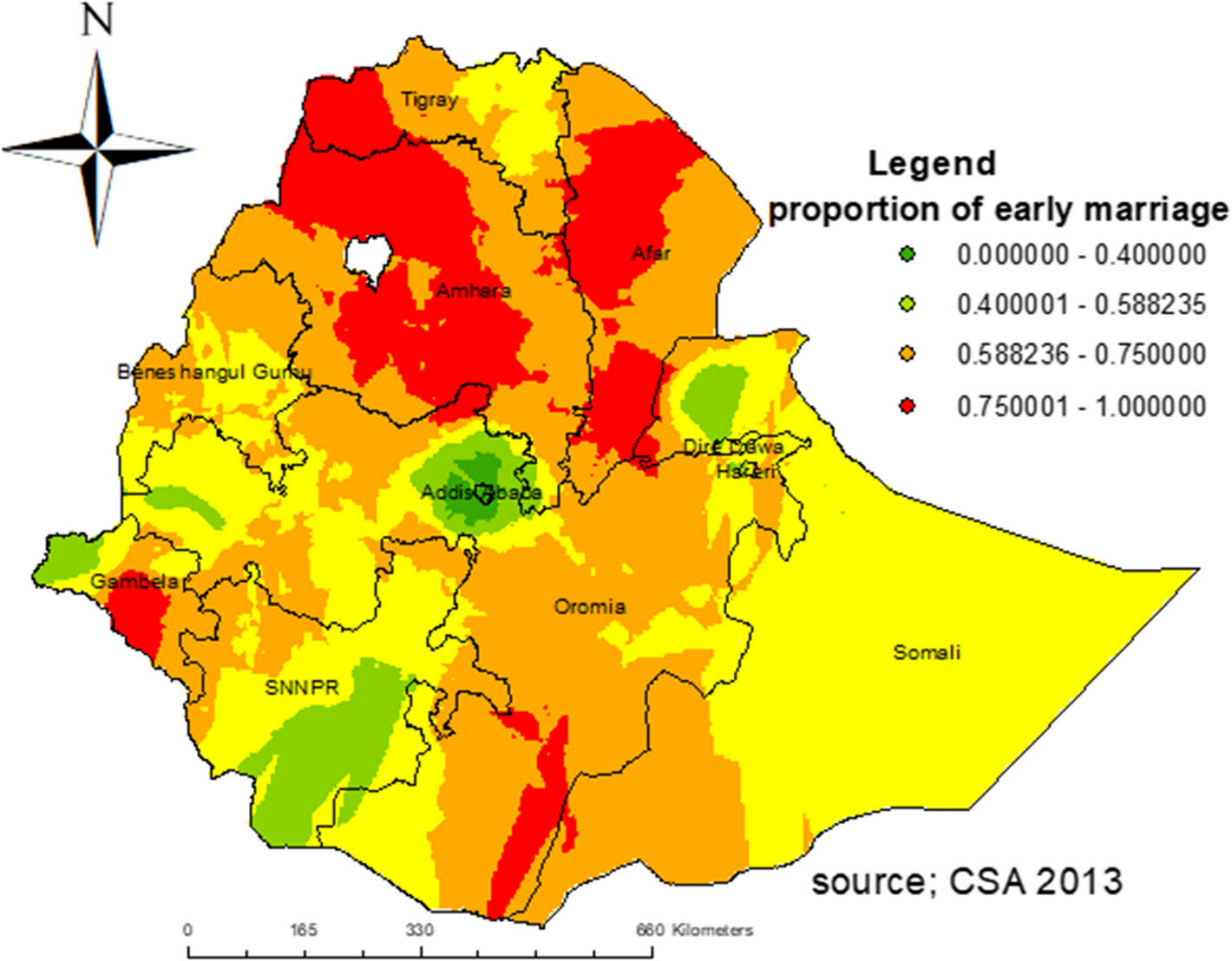
Interpolated special distribution of early marriage in Ethiopia, EDHS 2016

The Kriging interpolation predicted the highest prevalence of early marriage in the entire Amhara, south Oromia, west Tigray, entire Afar, and west Gambela regions. In contrast, the predicted lowest prevalence of early marriage was identified in Addis Ababa, Dire Dawa, the western part of Gambela, the eastern part of SNNPR, and eastern Oromia (**Fig. ****6**).

### Determinants of early marriage among women of a reproductive age

#### Random effect analysis

The results of the random-effects model revealed the presence of variation in early marriage prevalence across communities. The intra-cluster correlation coefficient in the null model indicated that 13% of the variation in early marriage was attributed to community-level factors. Moreover, the median odds ratio was 1.96 in the null model which indicates that early marriage was heterogeneous between clusters (EAs). In addition, as shown by PCV, about 56% of the variability in early marriage was explained by both individual and community level variables in the full model. Regarding model comparison/model fitness, the model with the lowest deviance (model 4) was the best-fitted model and we used this model to assess the determinants of early marriage among married women in Ethiopia (**Table ****2**).

**Table 2:** Multivariable multilevel logistic regression analysis for factors associated with early marriage among reproductive women in Ethiopian, EDHS 2016

Variables	Model 1	Model 2 (AOR with 95% CI)	Model 3 ( AOR with 95% CI)	Model 4 (AOR with 95% CI)
Level of women education				
Higher		1		1
Secondary		1.70 (1.29–2.25)		1.75 (1.32–2.33)
Primary		3.56 (2.69–4.71)		3.71 (2.80–4.92)
No education		3.99 (2.98–5.36)		4.21(3.13–5.66)
Working status of respondents				
Not working		1		1
working		1.03 (0.93–1.14)		1.05 (0.95–1.17)
Type of media exposed				
4		1		1
3		1.56 (0.99–2.50)		1.41 (0.23–2.28)
2		1.79 (1.13–2.82)		1.46 (0.93–2.32)
1		2.12 (1.34–3.36)		1.62 (1.00–2.59)
0		1.84 (1.16–2.92)		1.39 (0.87–2.23)
**Husband education**				
Higher		1		1
Secondary		1.18 (0.97–1.45)		1.22 (0.99–1.50)
Primary		1.30 (1.06–1.58)		1.35 (1.11–1.65)
No education		1.17 (0.95–1.44)		1.17 (0.95–1.44)
Wealth Index				
Poorest		1		1
Poorer		1.02 (0.88–1.19)		1.10 (0.94–1.28)
Middle		0.97 (0.83–1.15)		1.03 (0.87–1.23)
Richer		0.94 (0.79–1.11)		1.01 (0.84–1.21)
Richest		0.86 (0.73–1.02)		1.17(0.92–1.48)
**Decision on 1st marriage**				
Myself		1		1
Parents		2.06 (1.85–2.28)		1.88 (1.68–2.09)
Relatives/others		2.15 (1.60–2.90)		2.16 (1.60–2.91)
Residence				
Urban			1	1
Rural			1.62 (1.35–1.96)	1.23 (0.97–1.56)
Region				
Oromia			1	1
Amhara			1.76 (1.40–2.21)	1.77 (1.38–2.27)
Tigray			1.10 (0.87–1.39)	1.09 (0.84–1.40)
Somali			0.79 (0.62–1.01)	1.09 (0.84–1.42)
Afar			1.97 (1.52–2.56)	1.82 (1.37–2.42)
Benishangul-Gumuz			1.09 (0.85–1.40)	1.05 (0.81–1.37)
SNNPR			0.81 (0.65–1.01)	0.91 (0.72–1.14)
Gambella			1.06 (0.83–1.36)	1.44 (1.09–1.90)
Harari			0.83 (0.64–1.09)	1.06 (0.79–1.90)
Addis Ababa			0.42 (0.32–0.56)	0.50 (0.36–0.68)
Dire Dawa			0.98 (0.75–1.28)	1.01 (0.75–1.36)
Community poverty				
Low poverty			1	1
High poverty			1.04 (0.89–1.22)	1.06 (0.88–1.27)
Community women education				
low			1	1
High			1.08 (0.92–1.26)	0.96 (0.81–1.14)
Community husband education				
low			1	1
High			0.99 (0.84–1.16)	0.92 (0.77–1.09)
Community media exposure				
Low			1	1
High			1.01 (0.87–1.17)	1.08 (0.92–1.28)
**Random effects and model comparison**				
Community level variance (SE)	0.50 (0.05)	0.29 (0.04)	0.21 (0.03)	0.22 (0.03)
ICC (%)	13.2	8.1	6.0	6.3
Deviance (-2LL)	14,805.61	12,014.14	14,500.06	11,916.70
PCV (%)	Ref	42	58	56
MOR	1.96	1.67	1.54	1.56

#### Fixed effect analysis

In the multivariable multilevel logistic regression model educational status of women, decisions on first marriage, educational status of husband, and region were significantly associated with early marriage (*p *< 0.0.05).

The odds of early marriage among women with no education, primary and secondary education was 4.21(AOR = 4.21, 95% CI 3.13–5.66), 3.37 (AOR = 3.37, 95% CI 2.80–4.92), and 1.75 (AOR = 1.75, 95% CI 1.32–2.33) respectively, times higher as compared to women who completed higher education. The odds of early marriage among women whose first marriage decision was made by their parents and relatives were1.88 (AOR = 1.88, 95% CI 1.68–2.09), and 2.16 (AOR = 2.16, 95% CI 1.60–2.91) times higher as compared to those whose decision was made by themselves, respectively. Moreover, the odds of early marriage among women from Afar, Amhara, and, Gambela region was 1.82 (AOR = 1.82, 95% CI 1.37–2.42), 1.77 (AOR = 1.77, 95% CI 1.38–2.27), and 1.44 (AOR = 1.44, 95% CI 1.09–1.90) times higher as compared to women from the Oromia region respectively. In addition, the odds of early marriage among women from Addis Ababa was 50% (AOR = 0.50, 95% CI 0.36–0.68) lower as compared to women from Oromia region (**Table ****2**).

## Discussion

This study attempted to assess the spatial distribution and determinants of early marriage among ever-married women in Ethiopia using the national-level data. Since early marriage is highly related to economic growth [30], child and maternal health [16], exploring the spatial distribution of early marriage provides evidence for the need to target intervention programs in high-risk areas where early marriage is most likely to occur. Moreover, identifying risk areas and community-level determinants may minimize the cost of the interventions to implement [31].

In our study, the spatial distribution of early marriage was significantly varied across the country. The significant hotspot areas of early marriage were detected in Amhara, Afar, and Tigray regions. This finding is in line with other studies conducted in different countries, which pointed out the significant spatial variations of early marriage [32–34]. The geographical difference of early marriage across the regional states might be attributable to the regional variation of education among women and sociocultural differences related to early marriage. Our finding suggested regional differences in the educational status of women in Ethiopia with the lowest education attainment in the Afar region (only 1.35% of women had higher education attainment). The other study also indicated that increasing girls’ duration of schooling could possibly leads to a decline in early marriage [35]. It is true that non-educated women are less likely to be actively involved in different knowledge enhancement activities like reading materials, service promotions, and peer-discussions, which creates greater awareness of the harmful effects of early marriage. Moreover, marriage is a deep-rooted tradition in many Ethiopian communities. For example, in rural Amhara, there is a social and cultural belief that the presence of virginity before marriage is highly valued and unmarried girls whose aged greater than 14 years are usually stigmatized. Due to this, their daughters are forced to marry before they were 14 years old [36]. Additionally, in the Afar region, young women are less valued, have no control over resources, and have low decision-making power both at home and within the community, including their personal life choices. As a result of this rigid culture and tradition of marriage make young women forced to get married at early age [37]. Due to the limited resources and reduced capacity to protect their rights, the regional government of Afar has failed to respond to the needs of women [37]. Therefore, to eradicate early marriage by 2025 targeted intervention like enhancing the capacity to produce resources, encouraging women’s autonomy to participate in decision-making, and engaging different stakeholders with key expertise is recommended in hot spot areas. The multivariable model also revealed consistent findings in which Amhara, Afar, and Gambela were the places with the highest early marriage practice in Ethiopia.

In this study women’s education, power of decision on marriage, and region were significantly associated with early mirage.

Similar to many previous studies [18–22], in the current study, educated women were less likely to marry early compared to those relatively educated. This might be due to being educated changes people’s perceptions about what is an ideal age of first marriage, which creates greater awareness of the negative health consequences associated with early marriage and pregnancy such as fistula and an increased risk of maternal morbidity and mortality [38]. Education can play an important role in empowering girls and offering them alternative opportunities for the future [39]. Besides, the higher educational attainment the women had, the more knowledge women get about the best age for marriage, and the harmful health outcomes of early marriage [40, 41].

The odds of early marriage was higher among women whose first marriage decision was made by their parents and other relatives compared to those whose decision was made by the respondents themselves. This finding is in line with the finding in Ethiopia, in which more than 55% of the ever-married women have been pressured into marriage by their family [23]. This might be due to parents often feeling that a young girl is an economic burden. Thus, they believe that marrying their young daughters help to bring social as well as the financial benefits to the poor family [42]. Another justification might be that early marriage is deeply rooted in religious and cultural traditions of Ethiopian communities and this usually results in the early marriage of children without their consent and letting them decide on their own.

Our result also suggests that the odds of early marriage among women in Afar, Amhara, and Gambela region were higher as compared to women in the Oromia region. This finding is in line with the finding of other studies in Ethiopia [2, 14], but the odds of early marriage among women in Addis Ababa was lower as compared to women’s in Oromia region. This finding is consistent with the results of EDHS 2011 [14]. This might be due to culture, urbanization, and religious differences as well as disparities in the implementation of early marriage preventive actions across different regions of Ethiopia. Research findings also suggest that religious and cultural factors were associated with early marriage [43].

## Strength and limitation of the study

The main strength of this study is the use of nationally representative data, which was collected using standard and validated data collection tools. Additionally, we used an advanced model (multilevel analysis) that accounts for the correlated nature of the EDHS data in estimating the determinants factors with a combination of spatial analysis that allows the understanding of geographic variation in the occurrence of early marriage among reproductive-age women. However, this study is not free from limitations. Because of the secondary nature of the data, factors such as parents' knowledge of the best marital age and knowing someone who accused of early marriage have not been included in the analysis Besides, due to the cross-sectional nature of the data, we are also unable to show the cause and effect relationship between independent variables and early marriage. Recall bias may also be there and since the SaTScan analysis detects only circular clusters, irregularly shaped clusters might not be detected. Moreover, since this research included ever-married women aged 15–49 years compared with the Sustainable Development Goals (SDGs) indicator (20–24 years), the finding of this study may not be generalizable to the percentage of women aged 20 to 24 who married before the age of 18. Despite these limitations, this study's finding contributes to the existing literature by exploring the spatial pattern of early marriage and its determinants in Ethiopia which provides evidence of the need to target intervention programs in high-risk areas and populations.

## Conclusion

Early marriage was significantly varied in Ethiopia. The hotspot areas of early marriage were detected in Amhara, Tigray, and Afar Regions. Women with no formal education, women whose decision about first marriage was made by parents and relatives as well as women’s living in Amhara, Afar, and Gambela regions had an increased likelihood of early marriage. However, women who live in Addis Ababa had a decreased likelihood of early marriage. Therefore, targeting the early marriage policy interventions in those risk areas by focusing on the improvement of maternal education and the empowerment of women in decision-making could be vital to minimize and even to eliminate the early marriage habit in Ethiopia.

## Data Availability

All result based data were found in the manuscript and the datasets used and/or analyzed during the current study is available at https://www.dhsprogram.com.

## Abbreviations

AOR: Adjusted Odds Ratio
CI: Confidence Interval
EDHS: Ethiopian Demographic and Health Survey
ICC: Intra-cluster Correlation Coefficient
LLR: Log likelihood Ratio
MOR: Median Odds Ratio
PCV: Proportional Change in Variance
SNNPR: Southern Nations, Nationalities and Peoples' Region
